# Enhanced Efficiency of the Removal of Cytostatic Anthracycline Drugs Using Immobilized Mycelium of *Bjerkandera adusta* CCBAS 930

**DOI:** 10.3390/molecules26226842

**Published:** 2021-11-12

**Authors:** Kamila Rybczyńska-Tkaczyk

**Affiliations:** Department of Environmental Microbiology, The University of Life Sciences, Leszczyńskiego Street 7, 20-069 Lublin, Poland; kamila.rybczynska-tkaczyk@up.lublin.pl or teresa.kornilowicz@up.lublin.pl

**Keywords:** daunomycin, doxorubicin, mitoxantrone, versatile peroxidases, genotoxicity, biotoxicity

## Abstract

The aim of this study was to evaluate the bioremoval of anthracycline antibiotics (daunomycin-DNR, doxorubicin–DOX, and mitoxantrone-MTX) by immobilized mycelium of *B. adusta* CCBAS 930. The activity of oxidoreductases: versatile peroxidases (VP), superoxide dismutase (SOD), catalase (CAT), and glucose oxidase (GOX), and the levels of phenolic compounds (PhC) and free radicals (SOR) were determined during the biotransformation of anthracyclines by *B. adusta* strain CCBAS 930. Moreover, the phytotoxicity (*Lepidium sativum* L.), biotoxicity (MARA assay), and genotoxicity of anthracyclines were evaluated after biological treatment. After 120 h, more than 90% of anthracyclines were removed by the immobilized mycelium of *B. adusta* CCBAS 930. The effective biotransformation of anthracyclines was correlated with detoxification and reduced genotoxicity.

## 1. Introduction

Anthracyclines have been used for cancer treatment for over 50 years and are still included in the World Health Organization (WHO) model list of essential medicines [1]. Daunomycin (daunorubicin, DNR), doxorubicin (DOX), and mitoxantrone (MTX) are used to treat acute myeloid and lymphocytic leukemias, chronic myelogenous leukemia, Kaposi’s sarcoma, and breast cancer [2,3]. The effectiveness of these cytostatic drugs is related to their presence in the environment. Anthracyclines can enter the water system from various sources: via hospital wastewater, urine from patients, and as industrial waste [4,5,6]. Even in low concentrations, pharmaceuticals are cytotoxic, teratogenic, genotoxic, and carcinogenic [7,8]. Moreover, they can accumulate via biomagnification in aquatic and terrestrial organisms, or they can initiate a cascade of free radical reactions whose products are the highly toxic hydrogen peroxide and hydroxyl radical [9,10]. Currently, physical and chemical methods, e.g., ozonation, adsorption, membrane filtration, and electrolysis, are used to remove pharmaceuticals from the aquatic environment [2,11,12,13]. Given the health and environmental aspects, ecofriendly methods based on the use of microorganisms, especially filamentous fungi, are being proposed increasingly often [8,14,15]. In the environment, white rot fungi synthesize oxidoreductases, e.g., peroxidases, which facilitate the biodegradation of the lignocellulose complex of plant biomass. Due to their wide range of substrates, fungal peroxidases can decompose compounds with an aromatic structure, e.g., pesticides, melanoidin, industrial dyes, post-industrial lignin, personal care products, and pharmaceuticals [8,14,16,17,18,19,20,21,22,23].

Nevertheless, biological methods have some limitations. To eliminate the limitations of the use of microorganisms in bioremediation, i.e., their long cultivation time and the sensitivity of microorganisms to environmental factors, cultures of microorganisms should be modified. Immobilization is a technique that allows the complete or partial immobilization of microorganisms and provides them with free access to nutrients and discharge of metabolic products [24,25]. Trapping is a frequently used method for immobilization of mycelium. It consists of immobilizing the material with a carrier, e.g., sodium alginate, agar, pectin, chitosan, polyacrylamide, or epoxy resins. The most common method is immobilization with the use of sodium alginate. The advantages of immobilization include increased stability and prolongation of enzyme activity, as well as lowering the cost of the process [16].

Therefore, the aim of this study was to evaluate the efficiency of bioremoval and detoxification of anthracycline antibiotics by immobilized mycelium of the *B. adusta* strain CCBAS 930. The activity of oxidoreductases (i.e., peroxidases, superoxide dismutase, catalase and glucose oxidase) and the levels of free radicals and phenolic compounds during the biotransformation of DNR, DOX, and MTX were characterized. Moreover, bio-, phyto- and genotoxicity assays were performed to determine the toxicity of the drugs after fungal biotransformation. Since anthracyclines are characterized by a strong pro-oxidant capacity and can possibly produce phenolic acids during anthraquinone biotransformation, the oxidant properties of post-liquid cultures during treatment with immobilized mycelium of *B. adusta* CCBAS 930 were assessed.

## 2. Results

### 2.1. Efficiency of Anthracycline Removal Using Immobilized Mycelium of B. adusta CCAS 930

**Figure A1:**
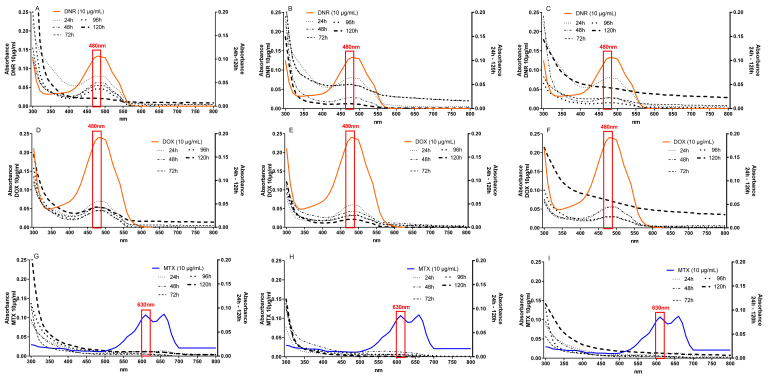
Anthracycline antibiotics (DNR—daunomycin, DOX—doxorubicin, MTX—mitoxantrone) removal using 4-week-old immobilized mycelium of *B. adusta* CCBAS 930 over cycle 1 (**A**,**D**,**G**), 2 (**B**,**E**,**H**) and 3 (**C**,**F**,**I**).

**Figure A2:**
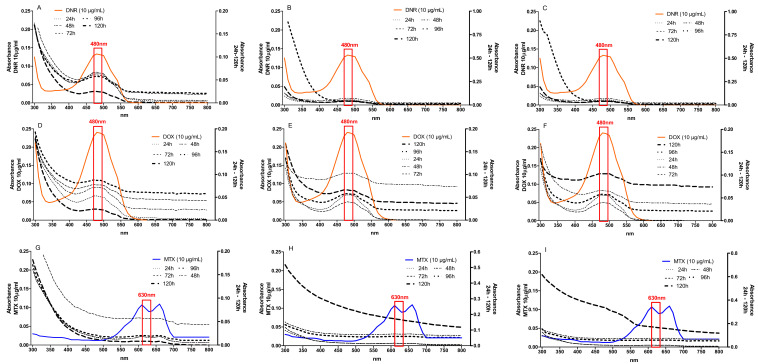
Anthracycline antibiotics (DNR—daunomycin, DOX—doxorubicin, MTX—mitoxantrone) removal using 12-week-old immobilized mycelium of *B. adusta* CCBAS 930 over cycle 1 (**A**,**D**,**G**), 2 (**B**,**E**,**H**) and 3 (**C**,**F**,**I**).

In order to check the efficiency of anthracycline removal and the impact of the immobilized mycelium storage conditions on the process efficiency, the experiment was carried out with the use of 4 and 12-week-old immobilized mycelium, which was stored in sterile 0.9% NaCl at 4 °C. The immobilized cultures of *B. adusta* CCBAS 930 were characterized by a significant decrease in the color of the medium supplemented with the anthracycline antibiotics (90% after 120 h). The effective removal of DNR, DOX, and MTX by the immobilized mycelium stored for 4 and 12 weeks at 4 °C was observed after 24 h, and the efficiency was 50–52%, 76.70–78.50%, and 83.63–92%, respectively. In the same culture conditions after 120 h, DNR, DOX, and MTX were removed with an efficiency of 81.20–92.50%, 72.50–87.20%, and 92.10–99.09%. In the next 2nd and 3rd cycles, the degree of DNR and DOX removal by the 4-week-old immobilized mycelium slightly decreased to 87.50–67.70% and 82–56.40% (Figure A1). In the case of the 12-week storage of the immobilized mycelium, the efficiency of the process decreased as well. The DNR and DOX removal rates during the 2nd and 3rd cycles of these conditions were in the range of 69.92–55.50% and 72.50–60%, respectively. Only for MTX was the removal efficiency using the 4 and 12 week-old immobilized mycelium at the same level (90–92%; Figure A2).

### 2.2. Activities of Oxidoreductases

**Figure 1:**
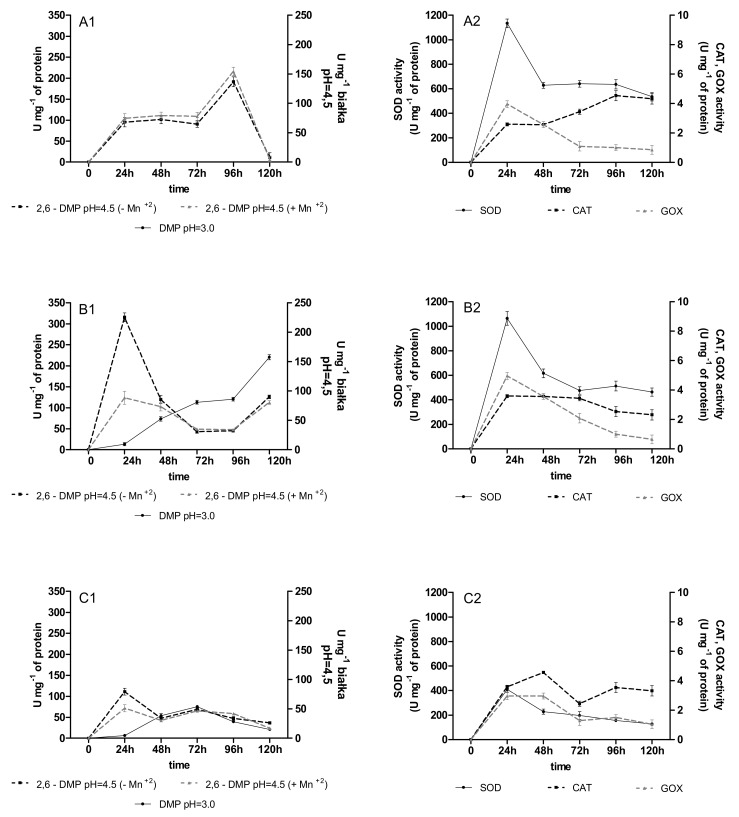
Activity of oxidoreductases (versatile peroxidase oxidized 2,6 DMP with/without Mn^+2^, GOX—glucose oxidase, CAT—catalase, SOD—superoxide dismutase) during anthracycline treatment ((**A1**,**A2**)—daunomycin, (**B1**,**B2**)—doxorubicin, (**C1**,**C2**)—mitoxantrone) by immobilized 4-week-old mycelium of *B. adusta* CCBAS 930 over 1 cycle.

**Figure 2:**
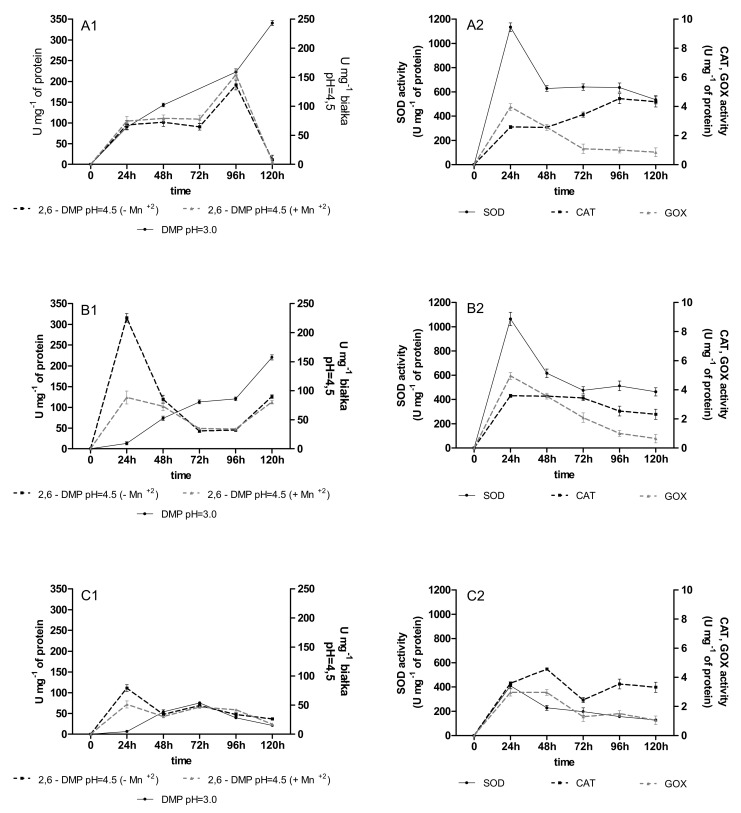
Activity of oxidoreductases (versatile peroxidase oxidized 2,6 DMP with/without Mn^+2^, GOX—glucose oxidase, CAT—catalase, SOD—superoxide dismutase) during anthracycline treatment ((**A1**,**A2**)—daunomycin, (**B1**,**B2**)—doxorubicin, (**C1**,**C2**)—mitoxantrone) by immobilized 12-week-old mycelium of *B. adusta* CCBAS 930 over 1 cycle.

**Figure A3:**
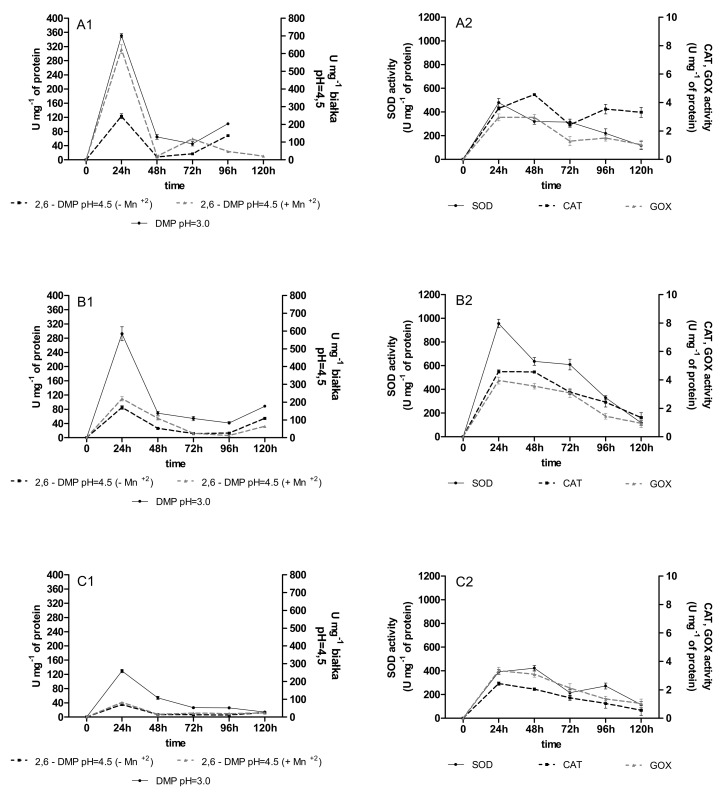
Activity of oxidoreductases (versatile peroxidase oxidized 2,6 DMP with/without Mn^+2^, GOX—glucose oxidase, CAT—catalase, SOD—superoxide dismutase) during anthracycline treatment ((**A1**,**A2**)—daunomycin, (**B1**,**B2**)—doxorubicin, (**C1**,**C2**)—mitoxantrone) by immobilized 4-week-old mycelium of *B. adusta* CCBAS 930 duringthe 2nd cycle.

**Figure A4:**
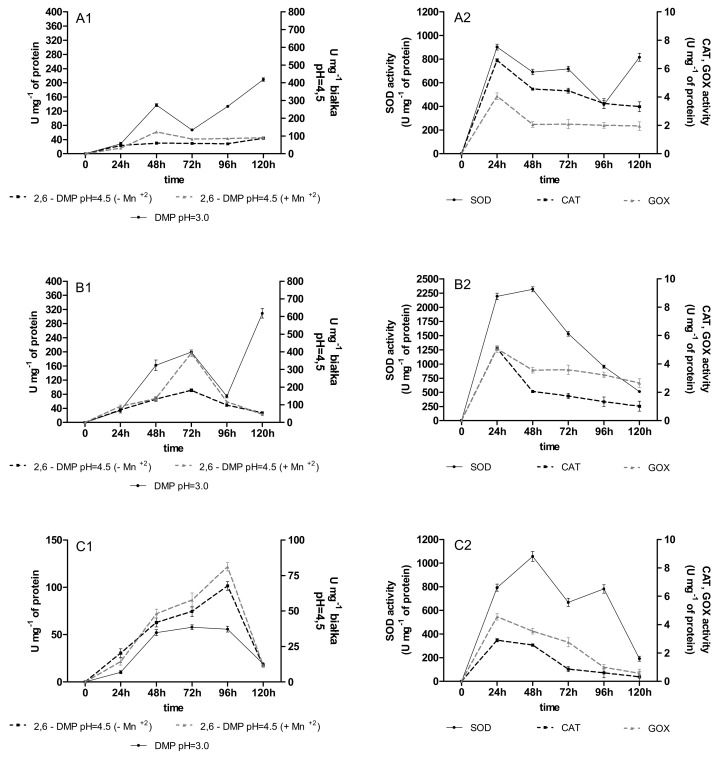
Activity of oxidoreductases (versatile peroxidase oxidized 2,6 DMP with/without Mn^+2^, GOX—glucose oxidase, CAT—catalase, SOD—superoxide dismutase) during anthracycline treatment ((**A1**,**A2**)—daunomycin, (**B1**,**B2**)—doxorubicin, (**C1**,**C2**)—mitoxantrone) by immobilized 12-week-old mycelium of *B. adusta* CCBAS 930 during the 2nd cycle.

**Figure A5:**
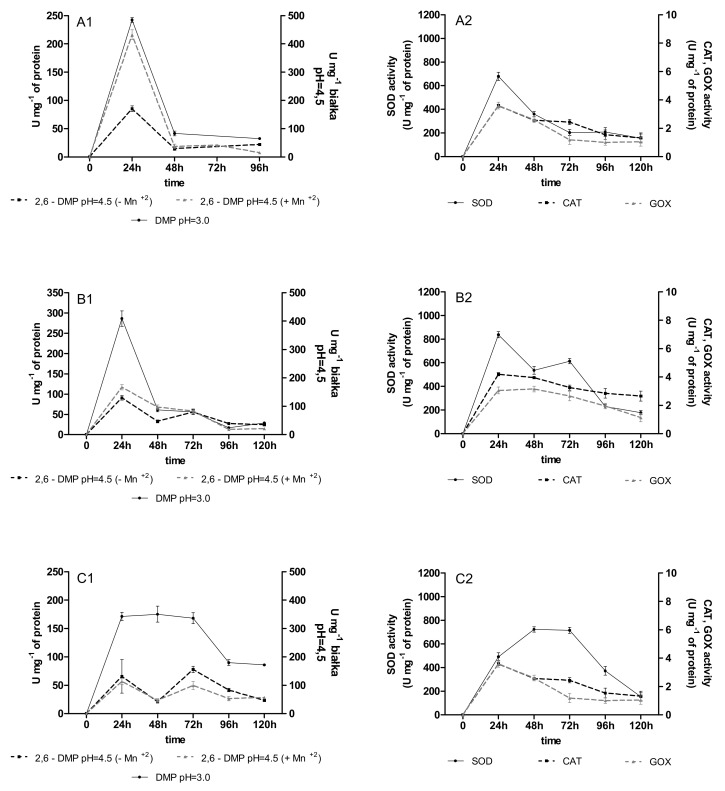
Activity of oxidoreductases (versatile peroxidase oxidized 2,6 DMP with/without Mn^+2^, GOX—glucose oxidase, CAT—catalase, SOD—superoxide dismutase) during anthracycline treatment ((**A1**,**A2**)—daunomycin, (**B1**,**B2**)—doxorubicin, (**C1**,**C2**)—mitoxantrone) by immobilized 4-week-old mycelium of *B. adusta* CCBAS 930 during the 3rd cycle.

**Figure A6:**
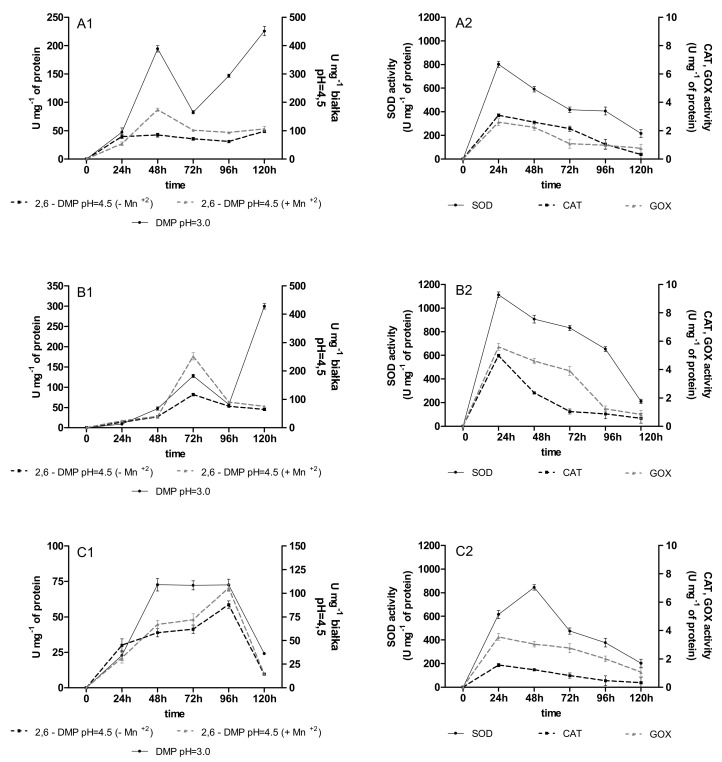
Activity of oxidoreductases (versatile peroxidase oxidized 2,6 DMP with/without Mn^+2^, GOX—glucose oxidase, CAT—catalase, SOD—superoxide dismutase) during anthracycline treatment ((**A1**,**A2**)—daunomycin, (**B1**,**B2**)—doxorubicin, (**C1**,**C2**)—mitoxantrone) by immobilized 12-week-old mycelium of *B. adusta* CCBAS 930 during the 3rd cycle.

The activity of versatile peroxidases (VP), SOD, CAT, and GOD was observed in the immobilized cultures of *B. adusta* CCBAS 930 with the addition of anthracyclines. The VP activity, especially measured at pH = 3.0, appeared during the first 24 h in the 4 and 12-week-old *B. adusta* CCBAS 930 immobilized cultures supplemented with all anthracyclines in all 1–3 cycles, and reached a maximum of 61–101 and 103–280 U/mg (Figure 1A1–C1). The activity of SOD, CAT, and GOD was also detected in the *B. adusta* CCBAS 930 cultures exposed to the anthracyclines. Maximum GOX and CAT activities were observed at 24 h (2.56–2.64 U/mg protein) and 72 h (5.44–6.44 U/mg protein). The highest SOD activity in the immobilized cultures of *B. adusta* CCBAS 930 supplemented with DNR, DOX, and MTX was noted during the first 24–48 h (480–1134 U/mg protein; Figure 2A2–C2). In the 2nd and 3rd cycle, for both the 4 and 12-week mycelium, the activity of oxidoreductases remained at a high level (Figure A3, Figure A4, Figure A5 and Figure A6).

### 2.3. Content of Phenolic Acids (PhC) and Free Radicals (SOR)

**Figure 3:**
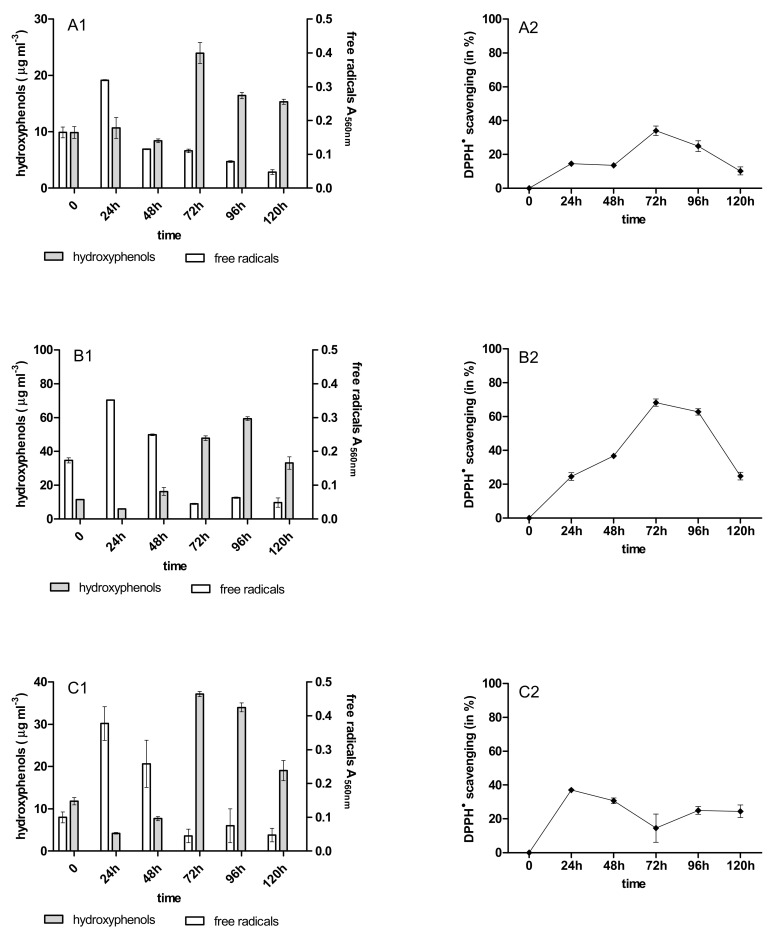
Content of hydroxyphenols (PCh), free radicals (SOR) and antioxidants activity during anthracycline treatment ((**A1**,**A2**)—daunomycin, (**B1**,**B2**)—doxorubicin, (**C1**,**C2**)—mitoxantrone) by immobilized 4-week-old mycelium of *B. adusta* CCBAS 930 over 1 cycle.

**Figure 4:**
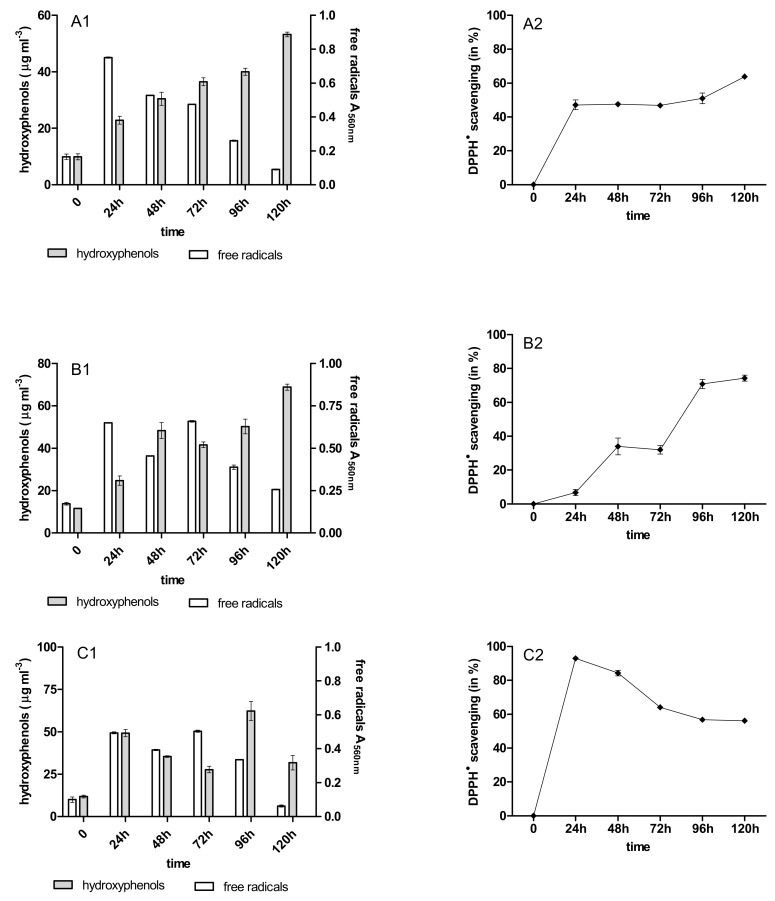
Content of hydroxyphenols (PCh), free radicals (SOR) and antioxidants activity during anthracycline treatment ((**A1**,**A2**)—daunomycin, (**B1**,**B2**)—doxorubicin, (**C1**,**C2**)—mitoxantrone) by immobilized 12-week-old mycelium of *B. adusta* CCBAS 930 over 1 cycle.

During the 120 h anthracycline biotreatment with the 4-week-old immobilized mycelium of *B. adusta* CCBAS 930, a systematic increase in the content of phenolic compounds and free radicals was observed up to 72 h. After this time, the content of phenolic compounds decreased to the value characteristic for the initial anthracycline solutions (Figure 3A1–C1). The increase in free radicals was observed after 24 h, then their content systematically decreased until the end of the cycle (Figure 3A2–C2). Using the 12-week-old immobilized mycelium, a significant increase in the content of PhC and SOR during their biotreatment was observed in all cytostatic variants (Figure 4A1,B2,C1). The content of phenolic compounds increased significantly in cultures with 12-week-old immobilized mycelium—especially for cultures with DNR, where in cycles 1–3 their content ranged from 23 to 63 mg/mL protocatechuic acid (Figure 3A1–C1, Figure A8A1–C1 and Figure A10A1–C1).

### 2.4. Antioxidant Activity of PhC after Anthracycline Biotreatment

**Figure A7:**
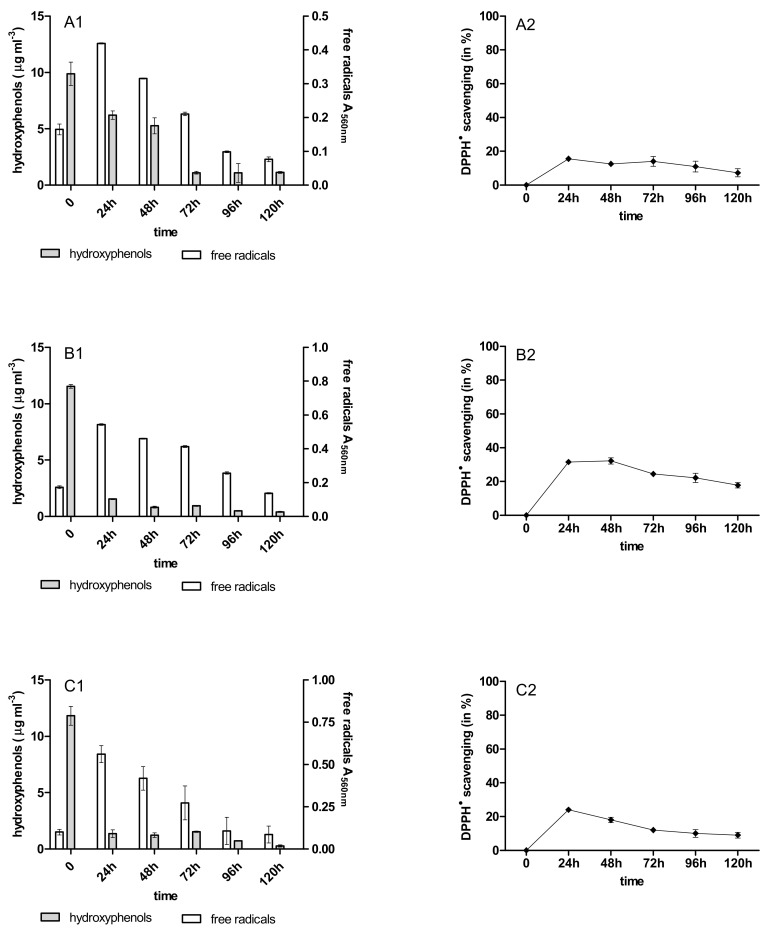
Content of hydroxyphenols (PCh), free radicals (SOR) (**A1**–**C1**) and antioxidants activity (**A2**–**C2**) during anthracyclines (daunomycin—(**A1**,**A2**), doxorubicin—(**B1**,**B2**) and mitoxantrone—(**C1**,**C2**)) treatment by immobilized 4-week-old mycelium of *B. adusta* CCBAS 930 duringthe 2nd cycle.

**Figure A8:**
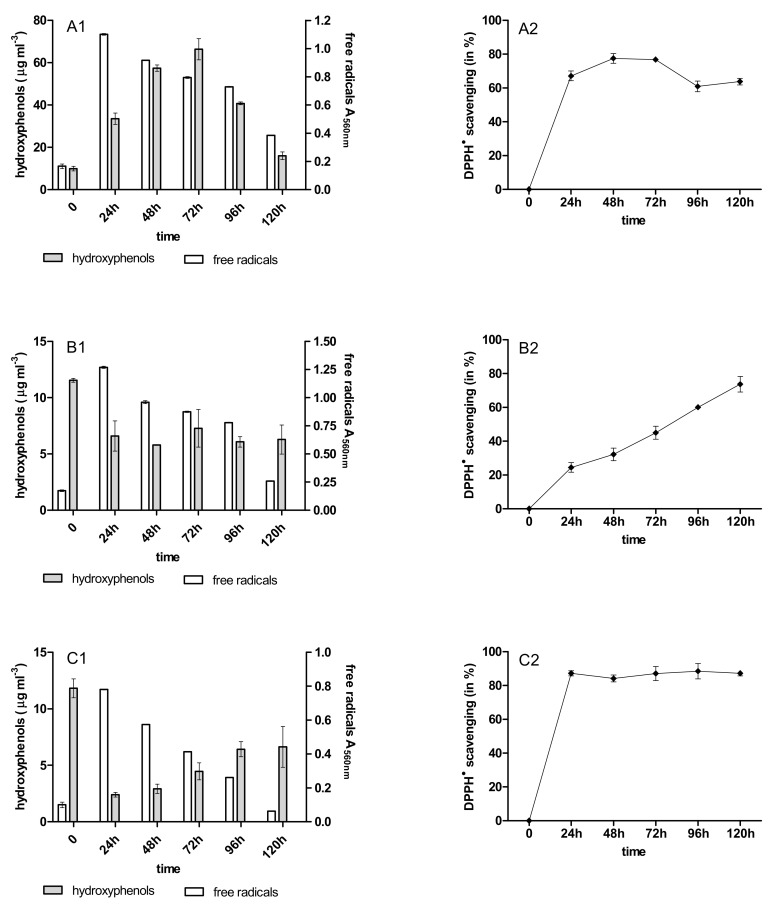
Content of hydroxyphenols (PCh), free radicals (SOR) (**A1**–**C1**) and antioxidants activity (**A2**–**C2**) during anthracyclines (daunomycin—(**A1**,**A2**), doxorubicin—(**B1**,**B2**) and mitoxantrone—(**C1**,**C2**)) treatment by immobilized 12-week-old mycelium of *B. adusta* CCBAS 930 during the 2nd cycle.

**Figure A9:**
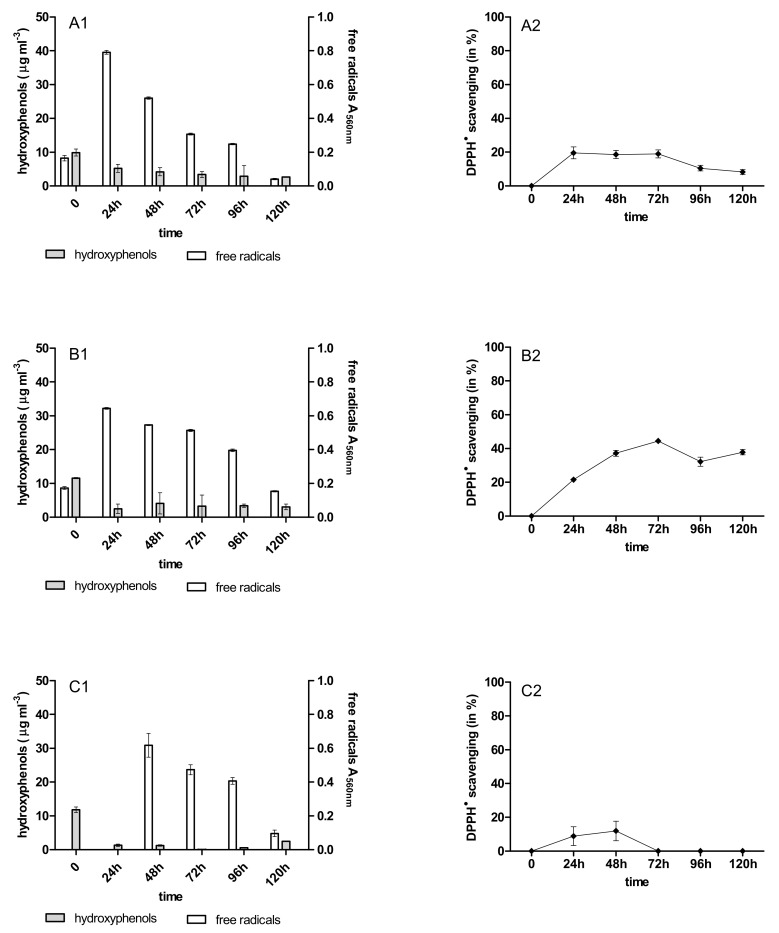
Content of hydroxyphenols (PCh), free radicals (SOR) (**A1**–**C1**) and antioxidants activity (**A2**–**C2**) during anthracyclines (daunomycin—(**A1**,**A2**), doxorubicin–(**B1**,**B2**) and mitoxantrone–(**C1**,**C2**)) treatment by immobilized 4 -week-old mycelium of *B. adusta* CCBAS 930 during the 3rd cycle.

**Figure A10:**
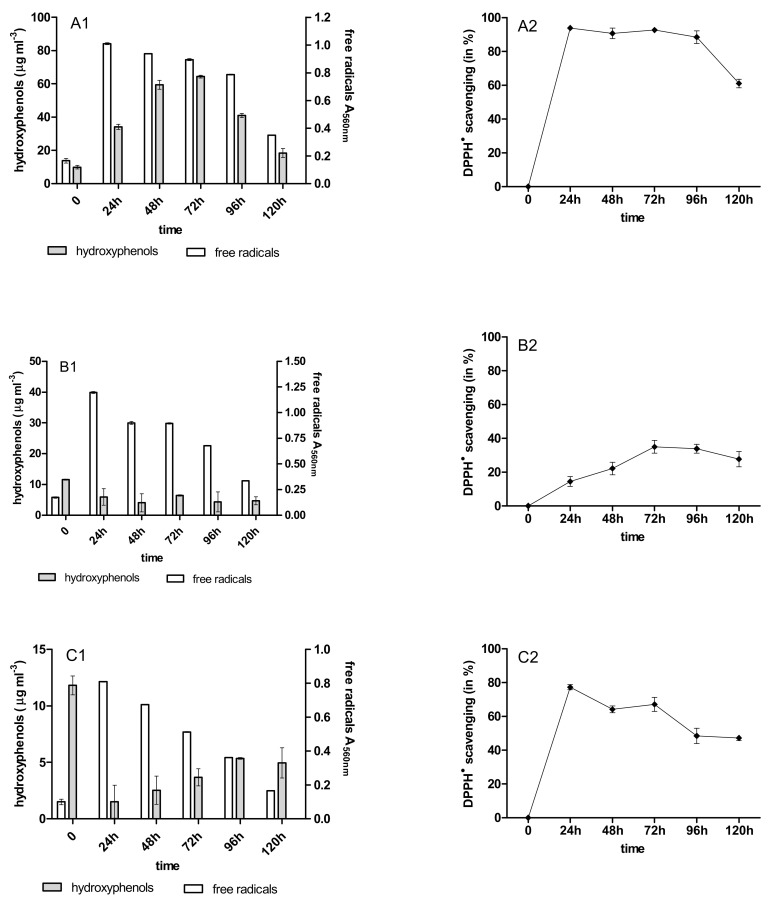
Content of hydroxyphenols (PCh), free radicals (SOR) (**A1**–**C1**) and antioxidants activity (**A2**–**C2**) during anthracyclines (daunomycin—(**A1**,**A2**), doxorubicin—(**B1**,**B2**) and mitoxantrone—(**C1**,**C2**)) treatment by immobilized 12-week-old mycelium of *B. adusta* CCBAS 930 during the 3rd cycle.

The initial solution of the anthracyclines and their biotransformation products were characterized by different anti-/pro-oxidative properties during the biotreatment with the immobilized mycelium of *B. adusta* CCBAS 930. The initial DNR, DOX, and MTX solutions (10 µg/mL) showed strong oxidative activity towards Trolox in the DPPH scavenging assay (data not shown). The antioxidant activity of the post-liquid cultures with DNR, DOX and MTX systematically increased in the 1st cycle *B. adusta* CCBAS 930 immobilized cultures, with a maximum DPPH^•^ scavenging activity of 30–70%, 20–70% and 14–37%, respectively (Figure 3A2–C2 and Figure 4A2–C2). The antioxidant properties in cycles 2 and 3 decreased in cultures with 4-week-old immobilized mycelium of *B. adusta* CCBAS 930 (Figure A7A2–C2 and Figure A9A2–C2). In the case of 12-week-old immobilized mycelium of *B. adusta* CCBAS 930 with DNR, DOX and MTX, DPPH^•^ scavenging activity increased over time, reaching values of 61–92%; 24–73% and 23–66%, respectively (Figure A8A2–C2 and Figure A10A2–C2).

### 2.5. Detoxification of Anthracyclines by Immobilized Mycelium of B. adusta CCBAS 930

**Figure 5:**
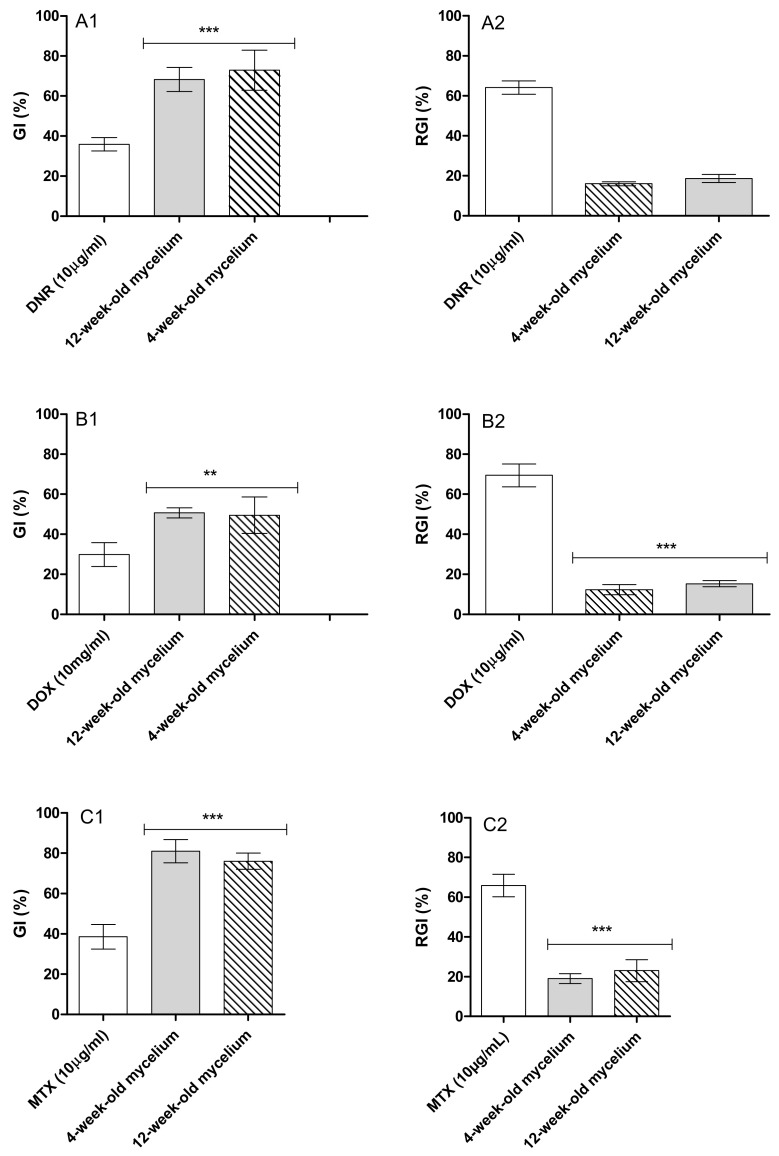
Phytotoxicity before and after 120 h treatment DNR, DOX and MTX (10 µg/mL) by immobilized mycelium of *B. adusta* CCBAS 930 after the 1st cycle for 4 and 12-week-old mycelium; GI—germination index (**A1**–**C1**), RGI—root growth inhibition (**A2**–**C2**); significantly different at *** *p* < 0.001, ** *p* < 0.01 compared with control (DNR, DOX and MTX 10 µg/mL).

**Figure 6:**
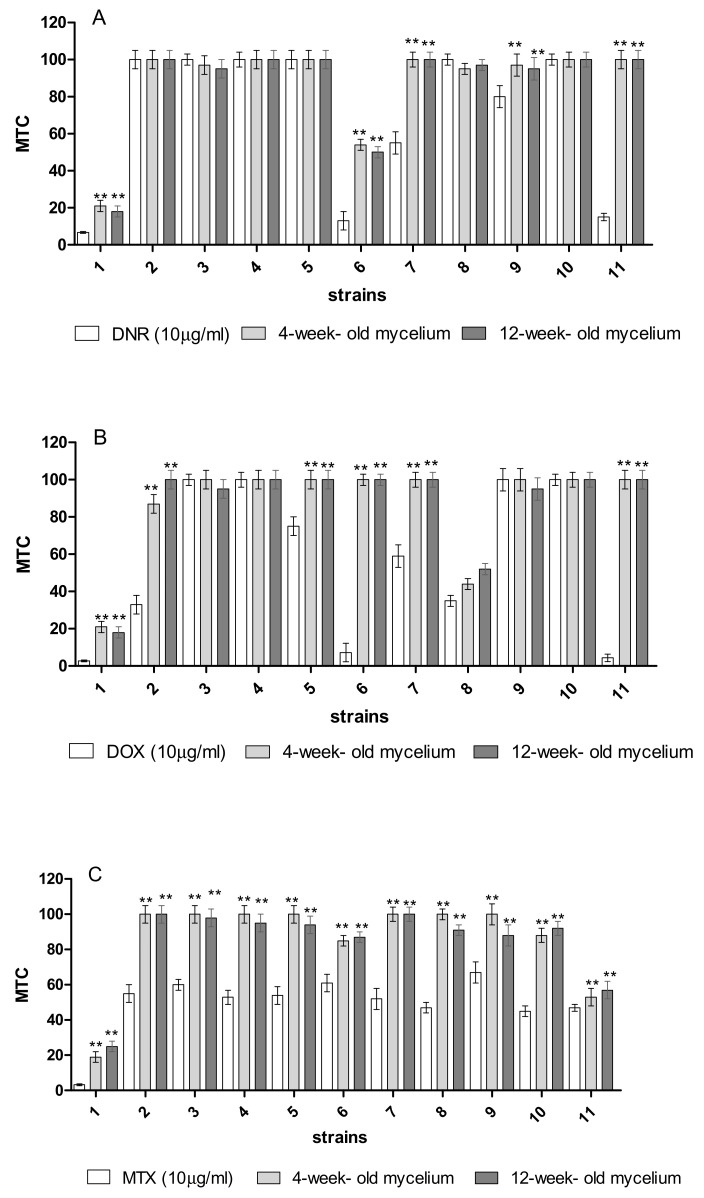
Microbial toxic concentration (MTC) value (% vol,) for each strain (**1**) *Microbacterium* sp., (**2**) *Brevundimonas diminuta*, (**3**) *Citrobacter freudii*, (**4**) *Comamonas testosteroni*, (**5**) *Entrococcus casseliflavus*, (**6**) *Delftia acidovorans*, (**7**) *Kurthia gibsoni*, (**8**) *Staphylococcus warneri*, (**9**) *Pseudomonas aurantiaca*, (**10**) *Serriatia rudidaea*, and (**11**) *Pichia anomala*, before and after 120 h treatment of DNR (**A**), DOX (**B**) and MTX (**C**) (µg/mL) by immobilized mycelium of *B. adusta* CCBAS 930 after the 3rd cycle for 4 and 12-old week mycelium; significantly different at ** *p* < 0.01.

Phyto-, bio-, and genotoxicity tests were used to assess the degree of anthracycline detoxification with the participation of the immobilized *B. adusta* CCBAS 930 mycelium. In the case of phytotoxicity towards *L. sativum* L., significant differences (*p* < 0.05) in the inhibition of seed germination were found before the DNR, DOX, and MTX treatments with the immobilized mycelium, i.e., GI = 35.85 ± 2.20, 29.88 ± 2.80, and 38.55 ± 6.05, respectively. After removal of anthracyclines using 4 and 12 week-old immobilized mycelium of *B. adusta* CCBAS 930, the germination index significantly increased (GI = 50–80; Figure 5A1–C1). In the case of root growth inhibition (RGI), significantly higher *L. sativum* L. root growth rates were also observed after the application of the treated post-liquid cultures (Figure 5A2–C2). The effectiveness of the biodegradation of anthracyclines was also assessed using the MARA assay. The toxic concentrations of the tested samples were evaluated for the most sensitive test organism (MTC min.% vol.) and for the most resistant test organism (MTC max.% vol.), and the average toxic concentration value was determined for all tested microorganisms. A wide range of sensitivities of the MARA species was noted before and after the treatment of anthracyclines by 4 and 12-week-old immobilized mycelium of *B. adusta* CCBAS 930, with the most sensitive species being *Microbacterium* sp.; MTC min. = 3.3, 2.7, and 6.7%, respectively. As a result of the biodegradation of the cytostatics by the immobilized mycelium, the average MTC values increased from 33–52% (initial solution of anthracyclines 10 µg/mL) to 65–82% (treatment with the immobilized mycelium). Based on the average MTC values, a significant decrease in DNR, DOX, and MTX toxicity was observed after biotreatment in many of the strains (Figure 6A–C).

The genotoxicity of anthracyclines after treatment with the immobilized mycelium of *B. adusta* CCBAS 930 was investigated using the SOS ChromoTest, based on *Escherichia coli* PQ37 bacteria. The effects were dose-dependent and decreased with increasing sample dilutions. Our results showed the highest genotoxicity of the initial solutions of DNR, DOX, and MTX with corrected induction factors (CIF) of 3.05, 2.50, and 2.20, respectively (data not shown). Thus, the results indicate that the anthracyclines before biodegradation were genotoxic at lower concentrations (2.5 and 1.25 µg/mL) and cytotoxic at higher concentrations (5 and 10 µg/mL). After the biotreatment, the CIF factor was <1.2, and the genotoxicity of the samples was on average 30–50% lower (Table 1). We did not observe any genotoxicity with metabolic activation (with the S9 fraction; data not shown).

## 3. Discussion

Removing xenobiotics from the environment is currently one of the most important challenges. Climate changes and decreasing drinking water resources mean that solutions are sought that are efficient and, at the same time, safe for the environment. In addition, treated post-industrial sewage, free of toxic substances, can be reused—for example in agricultural irrigation [6,26]. Cell-free enzymes in solution are poorly stable and their active sites can be inhibited. This increases the cost associated with this technology, since each enzymes can be used only once [7]. However, it is possible to increase the production, efficiency and stability of enzymes or mycelium by using immobilization, which in most cases also increases its regeneration [16,24,27,28]. Studies usually show an increase in the efficiency of xenobiotics removal with the use of immobilized peroxidases or laccases [29,30,31,32]. From the application point of view, obtaining immobilized mycelium is faster, easy for application and cheaper than immobilizing the enzyme [33]. Recent studies indicate that the immobilization of white rot fungi contributes to increases in the biosynthesis of oxidoreductases, e.g., peroxidases responsible for the biodegradation and detoxification of xenobiotics, e.g., melanoidin, textile wastewater and pharmaceutical compounds [16,33,34,35,36]. Immobilized mycelium of *B. adusta* CCBAS 930 was characterized by an overproduction of horseradish-like (HRP-like) and versatile (VP) peroxidase, which was associated with a 5-fold and 3-fold increase in the effectiveness of melanoid and anthraquinone dyes Alizarin Blue Black B and Acid Blue 129 removal—over 90% and 65.08–56.57% after 7 days, respectively [16,36]. Other studies have shown an increased activity of Mn-dependent peroxidase (MnP) and laccase (Lac) in the immobilized culture of *B. adusta*, and lignin degradation of 40%, and decolorization was about 70% with incubations of 40 h [37]. The research carried out in this study shows that immobilization of *B. adusta* CCBAS 930 significantly increases the efficiency of anthracycline removal too. Immobilization significantly increases the effectiveness of anthracycline removal, as evidenced by rates of over 90% of their removal after 72 h. Our previous study shows that to achieve 90% DNR and DOX removal (10 µg/mL), the stationary cultures of *B. adusta* CCBAS 930 strains should be grown for 21 days [8], e.g., immobilization shortens the time of anthracycline removal by about seven-fold. Moreover, the storage time does not affect the anthracycline removal efficiency in subsequent cycles. An additional advantage of mycelium immobilization is the overproduction of enzymes with high application potential [16,38]. Our research showed that over three cycles of anthracycline removal, the effectiveness of the process was at the same level (80–90%), even after 12 weeks of storage, in immobilized mycelium at 4 °C. Temperatures below the minimum usually inhibit microbial growth by slowing down metabolism, but do not kill the bacteria. Our previous research confirms that at 5 °C, the metabolism of *B. adusta* CCBAS 930 is slower, but its growth is not inhibited in the mineral medium [39]. The efficient removal of cytostatics in this study has been associated with the production of VP peroxidase. Earlier studies have shown the efficient removal of pharmaceuticals such as daunomycin, doxorubicin, diclofenac, sulfamethoxazole and naproxen with the participation of VPs [8,40]. Versatile peroxidases (VPs) are unique enzymes that exhibit lignin-degrading peroxidase (LiP) and MnP activity. Due to their activity over a wide range of temperatures (30–70 °C) and pH (3.0–7.0), VPs have been described as one of the most effective oxidoreductases for biotechnological applications—especially for bioremediation of industrial wastewater [41,42]. Among the different basidiomycete peroxidases, VPs present a particular interest due to their catalytic versatility, including the degradation of compounds that other peroxidases are not able to oxidize directly. VPs are able to oxidize different types of molecular structures such as low-and high-redox-potential dyes, and phenolic/non-phenolic compounds [43].

During anthracycline biotransformation by immobilized mycelium of *B. adusta* CCBAS 930 changes in phenolic acid production were observed. The content of phenolic compounds in the 1st round of the experiment, when the mycelium was stored for 4 weeks, was correlated with an increase in absorbance in the range of 240 nm–250 nm, which is the maximum absorbance for protocatechuic acid. In the next 2nd and 3rd cycles of the experiment, the storage time increased the content of phenolic compounds in post-culture fluids. The lack of correlations in the content of these compounds with absorbance increases at 240 nm–250 nm [44] suggested that their biosynthesis was associated with the metabolism of *B. adusta* CCBAS 930. Earlier studies have shown that fungi, e.g., *Bjerkandera adusta,* synthesize phenolic compounds [45,46], and that the addition of exogenic phenolic acids increases the efficiency of the production of phenolic compounds [47]. Moreover, during the biotransformation of anthraquinone derivatives by *B. adusta* CCBAS 930, an increase in the content of phenolic compounds was observed [8]. On the other hand, under oxidative stress conditions induced by pro-oxidants, e.g., anthracyclines and menadione white rot fungi, produced phenolic compounds and SOR, as well as peroxidases and antioxidant enzymes SOD and CAT [8,48]. Our previous study indicated that during anthracycline biotransformation in stationary cultures of *B. adusta* CCBAS 930, changes in the oxidative properties of phenols metabolites were observed [8]. In this study, the evaluation of the antioxidant properties of phenolic metabolites formed during the removal/biotransformation of anthracyclines showed an increase in the neutralization of free radicals in a DPPH assay. Recent studies have shown that phenolic compounds with antioxidant properties may be formed after the degradation of Kraft’s lignin and anthracycline antibiotics [8,49]. This is confirmed by the correlation (*p* < 0.05) between the increase in the level of phenolic compounds during the removal of anthracyclines by the immobilized mycelium of *B. adusta* CCBAS 930 and antioxidant activity. The production of SOR during anthracycline treatment by immobilized *B. adusta* CCBAS 930 was also observed. Free radicals are formed not only as a result of oxidative stress induced by xenobiotics, but also as a result of physiological changes. Biodegradation of xenobiotics is the result of glucose co-metabolism. In addition to peroxidases, auxiliary enzymes—such as SOD and CAT—play an important role in the biodegradation of xenobiotics, which regulate the level of free radicals [48,50]. Biodegradation of xenobiotics is the result of glucose co-metabolism. During the primary metabolism of white rot fungi, glucose is broken down, resulting in the formation of H_2_O_2_—which during secondary metabolism, is necessary for the initiation of extracellular peroxidase activity [50]. In the next step, excess H_2_O_2_, not used by peroxidases, is broken down into O_2_ and H_2_O with the participation of catalase. This process is confirmed by the correlation (*p* < 0.05) between the increase in the levels of SOR during the removal of anthracyclines by the immobilized mycelium of *B. adusta* CCBAS 930 and the higher activities of GOX, SOD and CAT.

From the application point of view, the removal of xenobiotics should be safe for the environment, and the products of their biodegradation/biotransformation should be non-toxic. Due to their cytotoxic properties, the presence of anthracycline antibiotics in the aquatic environment may pose a threat to other organisms, e.g., plants, and aquatic and soil biota [51,52]. Moreover, biodegradation of pharmaceuticals by oxidoreductases take place through different pathways and several intermediates and end-products are generated during the reaction. The effectiveness of the detoxification of xenobiotics using biological methods should be measured using various assays that show the spectrum of detoxification, e.g., phyto-, bio- and genotoxicity [53]. Moreover, in the case of filamentous fungi, particular attention should be paid to assessing toxicity during the biodegradation or biotransformation of aromatic compounds [53,54]. Anticancer drugs are designed to inhibit the growth of cancer cells.

One of the main aspects of efficient biodegradation of pharmaceuticals is decreasing phytotoxicity. Irrigation of land with ineffectively treated sewage containing residues of pharmaceuticals causes the accumulation of these substances in the soil, which then penetrate into plants [52]. Pharmaceuticals have the potential to alter plant physiology and key biochemical pathways [55]. Studies focusing on anthracycline antibiotics (daunomycin, doxorubicin) have shown their adverse effects on root growth and development and seed germination of *L. sativum* L. at concentrations of 10 µg/mL [8]. On the other hand, the natural anthracycline antibiotic Da2B, with the same structure as daunomycin, caused no phytotoxicity in pepper plants—even at concentrations of 500 µg/mL [56].The phytotoxicity assessment of supernatants obtained after immobilized culture of *B. adusta* CCBAS 930 with the addition of DNR, DOX and MTX showed a higher germination index and a lower root growth inhibition of *L. sativum* L.

During the growth of fungi, in the presence of these compounds, secondary metabolites may form, e.g., some phenolic compounds, organic acids, or compounds that inhibit the growth of microorganisms [53]. Research by Kim et al. (2000) showed that the anthracycline antibiotic Da2B at concentrations in the range of 7.5–10 µg/mL inhibited the growth of yeast *Saccharomyces cerevisiae* and *Bacillus subtilis* bacteria [56]. Other anticancer drugs (5-fluorouracil, irinotecan and oxaliplatin) are characterized by their antimicrobial activity against *B. subtilis* [57]. The research carried out in this study shows that during the biodegradation of anthracycline antibiotics by immobilized mycelium of *B. adusta* CCBAS 930, no toxic compounds for plants or microorganisms are formed. In addition, various molecules and metabolites are present in the environment that may interact with cytostatics, which may intensify the adverse effects of the substances, e.g., genotoxicity [58]. To assess the genotoxicity and mutagenicity of cytostatics, including anthracycline antibiotics, the most commonly used tests are: AMES, SOS Chromotest and comet assays [8,58,59,60,61]. The mutagenicity of anthracyclines may be explained by their intercalation of DNA, which prevents their replication and results in DNA damage and the binding of DNA-related enzymes such as topoisomerase II. Inhibition of topoisomerase generates oxidative stress, which contributes to the development of mutagenicity and genotoxicity [58,62]. Previous studies have indicated genotoxic effects of anthracyclines [8,58,61]. Zounková et al. (2007) [61] demonstrated the genotoxicity of doxorubicin using the SOS Chromotest, without and with metabolic activation at a concentration of 0.074 and 0.098 mg/L, respectively. Exposure to DOX, even at low concentrations of ≤0.05 µg/L, caused DNA damage in *Ceriodaphnia dubia* and *Daphnia magna* [58]. The research carried out in this study shows that the use of the immobilized mycelium of *B. adusta* CCBAS 930 to remove anthracycline antibiotics causes not only their detoxification, but also a decrease in genotoxicity.

## 4. Materials and Methods

### 4.1. Chemicals

Daunomycin hydrochlorine (≥90%), doxorubicin hydrochlorine (≥98%), mitoxantrone hydrochlorine (≥90%), 2.6-dimethoxyphenol (99%; 2.6-DMP), nitrotetrazolium blue (99%; NBT), protocatechuic acid (97%), sodium alginate, calcium chloride (93%), 30% hydrogen peroxide, malonic acid (99%), DPPH^•^ (2.2-diphenyl-1-picrylhydrazyl), Trolox (97%), and the glucose oxidase assay were purchased from Sigma-Aldrich (St. Louis, MO, USA). The catalase assay kit was purchased from Merck Millipore. All other chemicals and reagents were of analytical grade.

### 4.2. Cultures of B. adusta CCBAS 930

The anamorphic *B. adusta* strain CCBAS 930 was isolated from black earth soil (Pheozems, FAO) from a field near Lublin in south-eastern Poland (Korniłłowicz-Kowalska et al., 2006). The experiments were conducted in 100 mL of liquid mineral medium [23] supplemented with 0.25% glucose and DOX at a concentration of 10 µg/mL. After 10 days of visible growth of aerial mycelium of *B. adusta* CCBAS 930, the mycelium was separated from the supernatant and washed with sterile distilled water three times. The mycelium was used for immobilization.

### 4.3. Immobilization of B. adusta CCBAS 930 Mycelium

**Figure A11:**
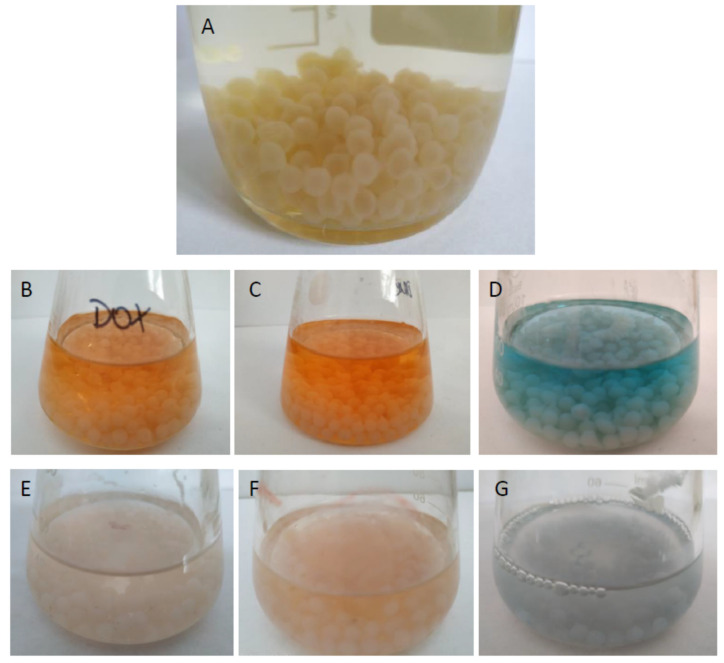
Immobilized mycelium of *B. adusta* CCBAS 930 (**A**) with anthracycline antibiotix: daunomycin (DNR) (**B**,**E**), doxorubicin (DOX) (**C**,**F**) and mitoxantrone (MTX) (**D**,**G**), before (**B**–**D**) and after 120 h treatment (**E**–**G**).

The immobilization of the *B. adusta* CCBAS 930 mycelium was performed using the Ca-alginate entrapment method proposed by Korniłłowicz-Kowalska and Rybczyńska-Tkaczyk (2020) [16]. The experiment was performed in agitation conditions (130 rpm/min, 120 h, 28 °C) using optimal volume (12.5 g) of immobilized mycelium for anthracyclines removal and 50 mL of mineral medium with 0.25% glucose and anthracycline antibiotics (10 µg/mL) (Figure A11).

### 4.4. Determination of Storage Conditions and Reusability of Immobilized B. adusta CCBAS 930 Mycelium

Storage stability was determined for 12 weeks when the immobilized mycelium of *B. adusta* CCBAS 930 was incubated in 0.9% NaCl at 4 °C. The decolorization experiment was conducted after 4 and 12 weeks. The reusability of the biodegradation of DNR, DOX, and MTX by the immobilized mycelium of *B. adusta* CCBAS 930 was monitored for up to 3 cycles. To evaluate the reusability of the immobilized mycelium, 50 mL of DNR, DOX, or MTX (10 µg/mL) in mineral medium were incubated for 5 days (120 rpm, 28 °C). Before using in the next cycle, the immobilized mycelium of *B. adusta* CCBAS 930 was washed three times with sterile 0.9% NaCl. For evaluation of the efficiency of anthracycline removal by the immobilized mycelium stored for 4 and 12 weeks, the decolorization degree, oxidoreductase activity, content of phenolic compounds and free radicals, and phyto-, bio, and genotoxicity were estimated.

### 4.5. Anthracycline Removal Using Immobilized Cells of B. adusta CCAS 930

The decolorization degree connected with the decomposition of chromophores was estimated by periodic absorbance at A480 nm measurements of clear post-culturing liquids with DNR and DOX and at A630 nm for MTX. Moreover, a visible spectrum in the wavelength range from 300 nm to 800 nm was measured during the treatment of the anthracyclines with the immobilized mycelium of *B. adusta* CCBAS 930.

### 4.6. Estimation of Oxidoreductase Activity

The activities of versatile peroxidases (VPs; Mn-dependent and Mn-independent activity) and superoxide dismutase (SOD) were evaluated using a microplate assay according to Rybczyńska-Tkaczyk et al. (2020) [23]. The activities of catalase (CAT) and glucose oxidase (GOX) were estimated using a catalase assay kit (Merc Millipore, Burlington, MA, USA) and a glucose oxidase assay kit (Sigma Aldrich), respectively. The GOX assay is based on D-glucose oxidation and production of H_2_O_2_, which reacts with the probe, generating a colorimetric (A570 nm) product proportional to the GOX activity. One unit of GOX is defined as the amount of the enzyme that generates 1 µmole of H_2_O_2_ per minute at 37 °C. The protein concentration was determined according to the Bradford method [63] using a protein assay kit (BioRad, Hercules, CA, USA).

### 4.7. Estimation of PhC and SOR

The content of PhC was determined at A740 nm [64] using a microplate assay with modifications [65]. The levels of SOR during anthracycline biotransformation by the immobilized mycelium of *B. adusta* CCBAS 930 were estimated based on the detection of superoxide-induced formazan formed from nitrotetrazolium blue (NBT) at A560 nm [66] Untreated medium with DNR, DOX, or MTX at a concentration of 10 µg/mL was used as a control.

### 4.8. Determination of the Antioxidative Activity of Initial Anthracycline Solutions and Post-Culture Fluids of B. adusta CCBAS 930

Antioxidant activity was measured using the DPPH^•^ scavenging assay according to Brand-Williams et al. (1995) [67]. Post-culture fluids (100 μL) and initial DNR, DOX, or MTX (10 µg/mL) samples were mixed with 100 μL of 25 mM DPPH• solution in 96% ethanol. After 30 min incubation at room temperature, sample absorbance was measured (A515 nm) using 96% ethanol as a blank sample. Trolox served as the positive control.

### 4.9. Estimation of the Toxicity of Anthracyclines in Immobilized Cultures of B. adusta CCBAS 930

#### 4.9.1. Phytotoxicity Assay

Phytotoxkit (Tigret, Poland) was used to determine the direct effects of the post-culture liquids of *B. adusta* CCBAS 930 after treatment with the immobilized mycelium (after 3 cycles for 4- and 12-week storage at 4 °C) of *B. adusta* CCBAS 930 on the germination and growth of young roots of *Lepidium sativum* L. in comparison to controls (distilled water) in a reference soil. The germination index (GI) and root growth inhibition (RGI) of seeds exposed to untreated and treated media were assessed and compared with germination and growth in the control.

#### 4.9.2. Multi-Species Microbial Assay (MARA)

The multi-species microbial assay (MARA) assay was applied to the post-culture liquids of *B. adusta* CCBAS 930 after the treatment with the immobilized mycelium (after 3 cycles for 4- and 12-week storage at 4 °C) of *B. adusta* CCBAS 930 according to the manufacturer’s protocol. The lyophilized microorganisms placed in row H of the microplates were rehydrated and pre-incubated for 4 h at 30 °C. A series of six dilutions of the initial solution of MTX (10 µg/mL) was placed in rows G–B of the microplates. The medium was introduced into row A (strain control). The microorganisms from row H were then added to each sample dilution. The microplate was incubated at 30 °C. After 18 h, the plates were scanned in a flatbed scanner (Epson Perfection V550 Photo). The results were processed using an image analysis program that facilitated calculation of the MTC (microbial toxic concentration) value [% vol.] for each strain.

#### 4.9.3. Genotoxicity Assay

The genotoxicity assay was performed using an SOS ChromoTest (distribution Tigret, Poland) according to the manufacturer’s protocol. Moreover, the S9 fraction (lyophilized rat liver with cytochrome P450 activity) was used to estimate the promutagenic potential of the tested compounds (after 1–3 cycles for 4- and 12-week storage of the mycelium at 4 °C) after the treatment with *B. adusta* CCBAS 930. Briefly, overnight bacterial cultures were grown in fresh LB medium to an optical density (OD600 nm) of 0.5–0.6, diluted 10-fold in double strength LB medium (20 g tryptone/L, 10 g yeast extract/L, 20 g sodium chloride/L, pH 7.4), and mixed (*v/v*) with the tested compounds, e.g., potential mutagens (or promutagens) and solvents. A negative control (distilled water) was always included in each assay. The bacteria were exposed to different initial concentrations of DOX, DNR, and MTX (10 µg/mL) and post-liquid cultures and incubated for 1.5 h at 37 °C. β galactosidase (β-gal) and alkaline phosphatase (AP) were assayed in 96-well plates. Significant genotoxic activity was defined as an adjusted induction factor (CIF) equal to or greater than 1.2.

### 4.10. Data Analysis

The data are presented as means ± standard deviation (SD) of three independent experiments. The data were analyzed using one-way analysis of variance (ANOVA) followed by Tukey’s multiple comparison procedure; *p*—probability value, *** *p* < 0.001, ** *p* < 0.01, and * *p* < 0.05 using STATISTICA v10.0 Software (StatSoft, Cracow, Poland).

## 5. Conclusions

Immobilization of the mycelium of the *B. adusta* CCBAS 930 strain significantly reduces the time required to remove anthracycline antibiotics to 120 h (over 90% removal). Based on this research, it can be concluded that the storage time of the immobilized mycelium of this strain can be up to 12 weeks, ensuring the same efficiency of the process. The production of VP does not change during the storage of the immobilized mycelium at 4 °C. However, the content of phenolic compounds and free radicals increases with the immobilized mycelium storage time. On the other hand, the evaluation of the antioxidant properties of phenolic metabolites formed during the removal/biotransformation of anthracyclines showed increased neutralization of free radicals in the DPPH assay. This may suggest the possibility of reusing phenolic compounds with antioxidant potential. Moreover, the studies showed a significant decrease in the phyto-, bio-, and genotoxicity of anthracycline antibiotics in the immobilized cultures of *B. adusta* CCBAS 930. More research is needed to provide the characteristics of VP produced by *B. adusta* CCBAS 930 and the possibility of its use in removing and detoxifying not only pharmaceuticals but also other environmentally hazardous xenobiotics.

## Figures and Tables

**Table 1:** Genotoxicity levels of anthracyclines after 3rd cycle treatment by 4-week-old immobilized mycelium of *B. adusta* CCBAS 930 (IM/Ba).

Samples	%vol.	CIF (4-Week Old Mycelium)	CIF (12-Week Old Mycelium)
DNR after treatment by IM/Ba	100	0.90 (±0.04)	1.02 (±0.03)
	50	0.67 (±0.05)	0.54 (±0.04)
	25	0.52 (±0.05)	0.51 (±0.04)
	12.50	0.12 (±0.02)	0.17 (±0.02)
	6.25	0.41 (±0.01)	0.32 (±0.01)
	3.12	0.13 (±0.02)	0.15 (±0.01)
	1.56	0.14 (±0.02)	0.16 (±0.01)
DOX after treatment by IM/Ba	100	0.90 (±0.04)	0.90 (±0.02)
	50	1.04 (±0.02)	1.02 (±0.02)
	25	1.09 (±0.02)	0.93 (±0.02)
	12.50	1.03 (±0.02)	0.85 (±0.02)
	6.25	0.48 (±0.01)	0.32 (±0.01)
	3.12	1.05 (±0.02)	0.56 (±0.02)
	1.56	1.03 (±0.06)	0.48 (±0.02)
MTX after treatment by IM/Ba	100	1.03 (±0.03)	0.76 (±0.04)
	50	0.42 (±0.03)	0.53 (±0.05)
	25	1.07 (±0.03)	0.75 (±0.05)
	12.50	1.04 (±0.02)	0.78 (±0.01)
	6.25	0.92 (±0.04)	0.80 (±0.04)
	3.12	0.94 (±0.02)	0.82 (±0.03)
	1.56	1.02 (±0.02)	0.32 (±0.02)

CIF—corrected induction factor, DNR—daunomycin, DOX—doxorubicin, MTX—mitoxantrone.

## Data Availability

All relevant data are included in the article.

## References

[1] McGowan J.V., Chung R., Maulik A., Piotrowska I., Walker J.M., Yellon D.M. (2017). Anthracycline Chemotherapy and Cardiotoxicity. Cardiovasc. Drugs Ther..

[2] Zhang J., Chang V.W.C., Giannis A., Wang J.Y. (2013). Removal of cytostatic drugs from aquatic environment: A review. Sci. Total Environ..

[3] Chugun A., Uchide T., Tsurimaki C., Nagasawa H., Sasaki T., Ueno S., Takagishi K., Hara Y., Temma K. (2008). Mechanisms responsible for reduced cardiotoxicity of mitoxantrone compared to doxorubicin examined in isolated guinea-pig heart preparations. J. Vet. Med. Sci..

[4] Lenz K., Mahnik S.N., Weissenbacher N., Mader R.M., Krenn P., Hann S., Koellensperger G., Uhl M., Knasmüller S., Ferk F. (2007). Monitoring, removal and risk assessment of cytostatic drugs in hospital wastewater. Water Sci. Technol..

[5] Mahnik S.N., Lenz K., Weissenbacher N., Mader R.M., Fuerhacker M. (2007). Fate of 5-fluorouracil, doxorubicin, epirubicin, and daunorubicin in hospital wastewater and their elimination by activated sludge and treatment in a membrane-bio-reactor system. Chemosphere.

[6] Malakar A., Snow D.D., Ray C. (2019). Irrigation water quality-A contemporary perspective. Water.

[7] Pereira C.S., Kelbert M., Daronch N.A., Michels C., de Oliveira D., Soares H.M. (2020). Potential of enzymatic process as an innovative technology to remove anticancer drugs in wastewater. Appl. Microbiol. Biotechnol..

[8] Rybczyńska-Tkaczyk K., Korniłłowicz-Kowalska T., Szychowski K.A. (2021). Possibility to Biotransform Anthracyclines by Peroxidases Produced by *Bjerkandera adusta* CCBAS 930 with Reduction of Geno- and Cytotoxicity and Pro-Oxidative Activity. Molecules.

[9] Jureczko M., Kalka J. (2020). Cytostatic pharmaceuticals as water contaminants. Eur. J. Pharmacol..

[10] Minotti G., Menna P., Salvatorelli E., Cairo G., Gianni L. (2004). Anthracyclines: Molecular advances and pharmacologie developments in antitumor activity and cardiotoxicity. Pharmacol. Rev..

[11] Jafarizad A., Rostamizadeh M., Zarei M., Gharibian S. (2017). Mitoxantrone removal by electrochemical method: A comparison of homogenous and heterogenous catalytic reactions. Environ. Health Eng. Manag..

[12] Stenglová-Netíková I.R., Petruzelka L., Stastny M., Stengl V. (2018). Anthracycline antibiotics derivate mitoxantrone—Destructive sorption and photocatalytic degradation. PLoS ONE.

[13] Kümmerer K. (2009). Antibiotics in the aquatic environment—A review—Part II. Chemosphere.

[14] Prieto A., Möder M., Rodil R., Adrian L., Marco-Urrea E. (2011). Degradation of the antibiotics norfloxacin and ciprofloxacin by a white-rot fungus and identification of degradation products. Bioresour. Technol..

[15] Muter O., Perkons I., Selga T., Berzins A., Gudra D., Radovica-Spalvina I., Fridmanis D., Bartkevics V. (2017). Removal of pharmaceuticals from municipal wastewaters at laboratory scale by treatment with activated sludge and biostimulation. Sci. Total Environ..

[16] Korniłłowicz-Kowalska T., Rybczyńska-Tkaczyk K. (2021). Decolorization and biodegradation of melanoidin contained in beet molasses by an anamorphic strain of Bjerkandera adusta CCBAS930 and its mutants. World J. Microbiol. Biotechnol..

[17] Zhang Y., Geißen S.U. (2010). In vitro degradation of carbamazepine and diclofenac by crude lignin peroxidase. J. Hazard. Mater..

[18] Varga B., Somogyi V., Meiczinger M., Kováts N., Domokos E. (2019). Enzymatic treatment and subsequent toxicity of organic micropollutants using oxidoreductases—A review. J. Clean. Prod..

[19] Taboada-Puig R., Lu-Chau T.A., Eibes G., Feijoo G., Moreira M.T., Lema J.M. (2015). Continuous removal of endocrine disruptors by versatile peroxidase using a two-stage system. Biotechnol. Prog..

[20] Torres E., Bustos-Jaimes I., Le Borgne S. (2003). Potential use of oxidative enzymes for the detoxification of organic pollutants. Appl. Catal. B Environ..

[21] Pan H., Xu X., Wen Z., Kang Y., Wang X., Ren Y., Huang D. (2017). Decolorization pathways of anthraquinone dye Disperse Blue 2BLN by Aspergillus sp. XJ-2 CGMCC12963. Bioengineered.

[22] Bergsten-Torralba L.R., Zamith H.P.S., Conde T.R., Aiub C.A.F., Felzenszwalb I., Da Silva M. (2016). Dye detoxification by Lentinula edodes INCQS 40220. Vigilância Sanitária Em Debate.

[23] Rybczyńska-Tkaczyk K., Korniłłowicz-Kowalska T., Szychowski K.A., Gmiński J. (2020). Biotransformation and toxicity effect of monoanthraquinone dyes during Bjerkandera adusta CCBAS 930 cultures. Ecotoxicol. Environ. Saf..

[24] Chapman J., Ismail A.E., Dinu C.Z. (2018). Industrial applications of enzymes: Recent advances, techniques, and outlooks. Catalysts.

[25] Sheldon R.A., van Pelt S. (2013). Enzyme immobilisation in biocatalysis: Why, what and how. Chem. Soc. Rev..

[26] Iglesias A., Garrote L. (2015). Adaptation strategies for agricultural water management under climate change in Europe. Agric. Water Manag..

[27] Dantas A., Valério A., Ninow J.L., de Oliveira J.V., de Oliveira D. (2019). Potential application of Thermomyces lanuginosus lipase (TLL) immobilized on nonporous polystyrene particles. Environ. Prog. Sustain. Energy.

[28] Ürek R.Ö., Pazarlioǧlu N.K. (2004). Purification and partial characterization of manganese peroxidase from immobilized *Phanerochaete chrysosporium*. Process Biochem..

[29] Rahmani K., Faramarzi M.A., Mahvi A.H., Gholami M., Esrafili A., Forootanfar H., Farzadkia M. (2015). Elimination and detoxification of sulfathiazole and sulfamethoxazole assisted by laccase immobilized on porous silica beads. Int. Biodeterior. Biodegrad..

[30] Bilal M., Adeel M., Rasheed T., Zhao Y., Iqbal H.M.N. (2019). Emerging contaminants of high concern and their enzyme-assisted biodegradation—A review. Environ. Int..

[31] Šekuljica N., Prlainović N., Jakovetić S.M., Grbavčić S., Ognjanović N.D., Knežević-Jugović Z.D., Mijin D. (2016). Removal of Anthraquinone Dye by Cross-Linked Enzyme Aggregates From Fresh Horseradish Extract. Clean Soil Air Water.

[32] Bilal M., Asgher M. (2015). Dye decolorization and detoxification potential of Ca-alginate beads immobilized manganese peroxidase. BMC Biotechnol..

[33] Arikan E.B., Isik Z., Bouras H.D., Dizge N. (2019). Investigation of immobilized filamentous fungi for treatment of real textile industry wastewater using up flow packed bed bioreactor. Bioresour. Technol. Rep..

[34] Akerman-Sanchez G., Rojas-Jimenez K. (2021). Fungi for the bioremediation of pharmaceutical-derived pollutants: A bioengineering approach to water treatment. Environ. Adv..

[35] Del Álamo A.C., Pariente M.I., Vasiliadou I., Padrino B., Puyol D., Molina R., Martínez F. (2018). Removal of pharmaceutical compounds from urban wastewater by an advanced bio-oxidation process based on fungi *Trametes versicolor* immobilized in a continuous RBC system. Environ. Sci. Pollut. Res..

[36] Rybczyńska-Tkaczyk K., Korniłłowicz-Kowalska T. (2020). Biodecolorization of anthraquinone dyes using immobilised mycelium of *Bjerkandera adusta* CCBAS930. E3S Web Conf..

[37] Nakamura Y., Sungusia M.G., Sawada T., Kuwahara M. (1999). Lignin-degrading enzyme production by *Bjerkandera adusta* immobilized on polyurethane foam. J. Biosci. Bioeng..

[38] Dosoretz C.G., Rothschild N., Hadar Y. (1993). Overproduction of lignin peroxidase by *Phanerochaete chrysosporium* (BKM-F-1767) under nonlimiting nutrient conditions. Appl. Environ. Microbiol..

[39] Korniłłowicz-Kowalska T., Rybczyńska-Tkaczyk K. (2020). Growth conditions, physiological properties, and selection of optimal parameters of biodegradation of anticancer drug daunomycin in industrial effluents by *Bjerkandera adusta* CCBAS930. Int. Microbiol..

[40] Eibes G., Debernardi G., Feijoo G., Moreira M.T., Lema J.M. (2011). Oxidation of pharmaceutically active compounds by a ligninolytic fungal peroxidase. Biodegradation.

[41] Sridhar M. (2016). Versatile Peroxidases: Super Peroxidases with Potential Biotechnological Applications—A Mini Review. J. Dairy Vet. Anim. Res..

[42] Taboada-Puig R., Lú-Chau T., Moreira M.T., Feijoo G., Martínez M.J., Lema J.M. (2011). A new strain of *Bjerkandera* sp. production, purification and characterization of versatile peroxidase. World J. Microbiol. Biotechnol..

[43] Ruiz-Dueñas F.J., Morales M., García E., Miki Y., Martínez M.J., Martínez A.T. (2009). Substrate oxidation sites in versatile peroxidase and other basidiomycete peroxidases. J. Exp. Bot..

[44] Gutierrez-Zetina S.M., Gonzalez-Manzano S., Perez-Alonso J.J., Gonzalez-Paramas A.M., Santos-Buelga C., Pellati F., Mercolini L., Sardella R. (2019). Preparation and Characterization of Protocatechuic Acid Sulfates. Molecules.

[45] Soliman E.R.S., El-Sayed H. (2021). Molecular identification and antimicrobial activities of some wild Egyptian mushrooms: *Bjerkandera adusta* as a promising source of bioactive antimicrobial phenolic compounds. J. Genet. Eng. Biotechnol..

[46] Nowacka N., Nowak R., Drozd M., Olech M., Los R., Malm A. (2015). Antibacterial, Antiradical Potential and Phenolic Compounds of Thirty-One Polish Mushrooms. PLoS ONE.

[47] Kulik T., Stuper-Szablewska K., Bilska K., Bu’skobu’sko M., Ostrowska-Kołodziejczak A., Załuski D., Perkowski J. (2017). Trans-Cinnamic and Chlorogenic Acids Affect the Secondary Metabolic Profiles and Ergosterol Biosynthesis by *Fusarium culmorum* and *F. graminearum* Sensu Stricto. Toxins.

[48] Jaszek M., Kos K., Matuszewska A., Grąz M., Stefaniuk D., Osińska-Jaroszuk M., Prendecka M., Jóźwik E., Grzywnowicz K., Jaszek M. (2014). Effective Stimulation of the Biotechnological Potential of the Medicinal White Rot Fungus: *Phellinus pini* by Menadione-Mediated Oxidative Stress. Appl. Biochem. Biotechnol..

[49] Alzagameem A., El Khaldi-Hansen B., Büchner D., Larkins M., Kamm B., Witzleben S., Schulze M., Xu C., Paleologou M. (2018). Lignocellulosic Biomass as Source for Lignin-Based Environmentally Benign Antioxidants. Molecules.

[50] Belinky P.A., Flikshtein N., Dosoretz C.G. (2006). Induction of lignin peroxidase via reactive oxygen species in manganese-deficient cultures of Phanerochaete chrysosporium. Enzym. Microb. Technol..

[51] Li D., Chen H., Liu H., Schlenk D., Mu J., Lacorte S., Ying G.G., Xie L. (2021). Anticancer drugs in the aquatic ecosystem: Environmental occurrence, ecotoxicological effect and risk assessment. Environ. Int..

[52] Gworek B., Kijeńska M., Wrzosek J., Graniewska M. (2021). Pharmaceuticals in the Soil and Plant Environment: A Review. Water Air Soil Pollut..

[53] Rybczyńska-Tkaczyk K., Święciło A., Szychowski K.A., Korniłłowicz-Kowalska T. (2018). Comparative study of eco- and cytotoxicity during biotransformation of anthraquinone dye Alizarin Blue Black B in optimized cultures of microscopic fungi. Ecotoxicol. Environ. Saf..

[54] Pozdnyakova N., Dubrovskaya E., Chernyshova M., Makarov O., Golubev S., Balandina S., Turkovskaya O. (2018). The degradation of three-ringed polycyclic aromatic hydrocarbons by wood-inhabiting fungus *Pleurotus ostreatus* and soil-inhabiting fungus *Agaricus bisporus*. Fungal Biol..

[55] Fu Q., Malchi T., Carter L.J., Li H., Gan J., Chefetz B. (2019). Pharmaceutical and Personal Care Products: From Wastewater Treatment into Agro-Food Systems. Environ. Sci. Technol..

[56] Kim B.S., Moon S.S., Hwang B.K. (2000). Structure elucidation and antifungal activity of an anthracycline antibiotic, daunomycin, isolated from *Actinomadura roseola*. J. Agric. Food Chem..

[57] Sangnier M., Bouguéon G., Berroneau A., Dubois V., Crauste-Manciet S. (2020). Removal of bacterial growth inhibition of anticancer drugs by using complexation materials. Pharm. Technol. Hosp. Pharm..

[58] Parrella A., Lavorgna M., Criscuolo E., Russo C., Isidori M. (2015). Eco-genotoxicity of six anticancer drugs using comet assay in daphnids. J. Hazard. Mater..

[59] Jolibois B., Guerbet M. (2006). Hospital wastewater genotoxicity. Ann. Occup. Hyg..

[60] Kümmerer K., Balcerzak W., Rezka P., Szuławska A., Czyż M., Doddapaneni H., Subramanian V., Fu B., Cullen D., Zounková R. (2013). Microbial cytochromes P450: Biodiversity and biotechnology. Where do cytochromes P450 come from, what do they do and what can they do for US?. Sci. Total Environ..

[61] Zounková R., Odráska P., Dolezalová L., Hilscherová K., Marsálek B., Bláha L. (2007). Ecotoxicity and genotoxicity assessment of cytostatic pharmaceuticals. Environ. Toxicol. Chem..

[62] Kupczewska-Dobecka M. (2020). Doxorubicine and doxorubicine hydrochloride–inhalable fraction. Documentation of proposed values of occupational exposure limits (OELs). Podstawy i Metod. Oceny Sr. Pr..

[63] Bradford M.M. (1976). A rapid and sensitive method for the quantitation of microgram quantities of protein utilizing the principle of protein-dye binding. Anal. Biochem..

[64] Singleton V.L., Rossi J.A. (1965). Colorimetry of Total Phenolics with Phosphomolybdic-Phosphotungstic Acid Reagents. Am. J. Enol. Vitic..

[65] Święciło A., Rybczyńska-Tkaczyk K., Najda A., Krzepiłko A., Prażak R., Zawiślak G. (2018). Application of growth tests employing a Δsod1 mutant of Saccharomyces cerevisiae to study the antioxidant activity of berry fruit extracts. LWT.

[66] Paździoch-Czochra M., Malarczyk E., Sielewiesiuk J. (2003). Relationship of demethylation processes to veratric acid concentration and cell density in cultures of *Rhodococcus erythropolis*. Cell Biol. Int..

[67] Brand-Williams W., Cuvelier M.E., Berset C. (1995). Use of a free radical method to evaluate antioxidant activity. LWT—Food Sci. Technol..

